# Multiomics analysis of human serum and animal experiments reveals the protective mechanism of Qingre Huoxue Decoction against rheumatoid arthritis

**DOI:** 10.3389/fimmu.2025.1526110

**Published:** 2025-03-07

**Authors:** Fuyuan Zhang, Congmin Xia, Guang Yang, Biyue Shang, Guangrui Huang, Kai Yuan, Hesong Wang, Xun Gong, Quan Jiang

**Affiliations:** ^1^ Guang’anmen Hospital, China Academy of Chinese Medical Sciences, Beijing, China; ^2^ Department of Chinese and Western Medicine, Peking University First Hospital, Beijing, China; ^3^ School of Life Sciences, Beijing University of Chinese Medicine, Beijing, China

**Keywords:** rheumatoid arthritis, bone destruction, proteomics, metabolomics, Chinese medicine, FBP1, AMPK

## Abstract

**Objective:**

Qingre Huoxue Decoction (QRHXD) is a traditional Chinese herbal prescription widely used in clinical practice with significant therapeutic effects on RA; however, its mechanism of action remains unclear. This study aimed to investigate the efficacy and underlying mechanisms of QRHXD in treating RA through clinical research, multiomics approaches, and animal experiments.

**Methods:**

We conducted a 24-week clinical study in which QRHXD was the primary treatment, collecting serum samples from patients before and after treatment for integrated proteomic and metabolomic analysis to identify potential therapeutic targets. Bioinformatics analysis of differentially expressed proteins (DEPs) and differential metabolites (DMs) was performed using hierarchical clustering, volcano plots, heat maps, Gene Ontology (GO), and Kyoto Encyclopaedia of Genes and Genomes (KEGG) analysis. To validate the identified therapeutic targets, we constructed a collagen-induced arthritis (CIA) mouse model.

**Results:**

Clinical research has shown that QRHXD can improve clinical symptoms and relevant indicators in RA patients, including the disease activity score-28 (DAS28), C-reactive protein (CRP), erythrocyte sedimentation rate (ESR), tender joint count (TJC), swollen joint count (SJC), visual analogue scale (VAS), patient-reported outcome (PRO), and health assessment questionnaire (HAQ). Proteomics and metabolomics analysis identified 83 DEPs and 54 DMs, including 46 upregulated and 37 downregulated proteins, as well as 11 upregulated and 43 downregulated metabolites. KEGG enrichment analysis revealed that DEPs are primarily associated with fatty acid degradation, ferroptosis, glycerolipid metabolism, and related pathways. The identified DMs are primarily associated with the AMPK signalling pathway, FoxO signalling pathway, glycolysis/gluconeogenesis, MTOR signalling pathway, and so on. GO enrichment analysis indicated that the DEPs were mainly associated with apoptotic mitochondrial changes, protein modification processes, fatty-acyl-CoA binding, and so on. Integrated proteomics and metabolomics analyses revealed a significant increase in fructose-1,6-biphosphatase 1 (FBP1) levels and a reduction in AMP-activated protein kinase (AMPK) levels in patients with RA. QRHXD inhibited FBP1 and activated AMPK signalling. Animal experiments validated the findings from proteomics and metabolomics analyses, demonstrating that QRHXD could also delay bone destruction and reduce inflammatory factor levels in CIA mice.

**Conclusion:**

QRHXD may reduce the disease activity of RA, attenuate the inflammatory response, and delay bone destruction by inhibiting FBP1 and activating the AMPK signalling pathway.

## Graphical Abstract

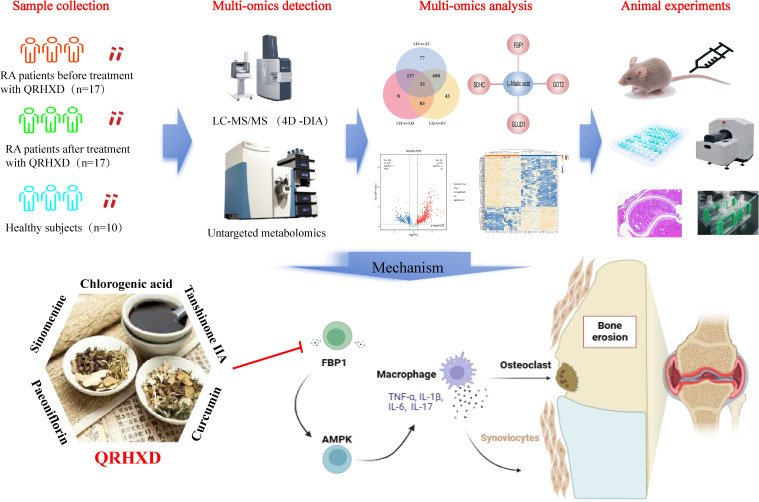

## Highlights

1. QRHXD can reduce disease activity and inhibit inflammatory response in RA patients.
2. High expression of FBP1 may be a key factor of high disease activity in RA.
3. QRHXD could modulate FBP1/AMPK signal pathway against RA.

## Introduction

Rheumatoid arthritis (RA) is a chronic, systemic autoimmune disease characterised by pathological changes such as synovitis and vascular opacification (1). With a global prevalence of 0.46% and an increasing annual incidence, RA has become a major cause of disability (2). Studies have shown that erosive joint changes are present in more than two-thirds of RA patients (3), while complications such as cardiovascular disease and interstitial lung disease significantly increase mortality (4). Early intervention and targeted treatment are necessary for controlling the disease progression, delaying bone destruction, and reducing complications (5).

Currently, the main treatments for RA include nonsteroidal anti-inflammatory drugs (NSAIDs), disease-modifying antirheumatic drugs (DMARDs), biologics, and glucocorticoids, which help slow disease progression, minimise tissue damage, and improve function (6). However, these drugs do not respond adequately in some patients and make treatment more difficult due to adverse effects (7). For example, methotrexate (MTX) is used as a first-line drug for the treatment of RA; however, up to 50% of patients treated with MTX do not achieve clinically satisfactory outcomes (8). TNF inhibitors such as etanercept, infliximab, and adalimumab are also widely used biologics for RA, and 30%–40% of RA patients do not achieve satisfactory outcomes (9). Therefore, it is essential to develop new and effective therapeutic strategies and medications to treat RA and reduce side effects.

Our team in China conducted a multicentre, 10,000-person prospective cohort study on RA, revealing that Chinese medicine (CM) can be used throughout the entire course of RA and exerts therapeutic effects (10). Qingre Huoxue Decoction (QRHXD), a traditional Chinese herbal prescription widely used in clinical practice, has demonstrated significant therapeutic effects on RA. QRHXD comprises 12 herbs: *Atractylodes chinensis* (Atractylodis Rhizoma), *Phellodendron amurense* (Phellodendri Chinensis Cortex), *Smilax glabra* Roxb (Smilacis glabrae rhizoma), *Lonicera japonica* Thunb (Lonicerae Japonicae Flos), *Astragalus membranaceus* (Fisch.) Bge. (Astragali Radix), *Paeonia lactiflora* Pall (Paeoniae Radix Rubra), *Dioscorea futschauensis* Uline ex R. Kunth (Dioscoreae Spongiosae Rhizoma), *Salvia miltiorrhiza* Bge. (Salviae Miltiorrhizae Radix Et Rhizoma), *Curcuma phaeocaulis* Val. (Curcumae Rhizoma), *Sinomenium acutum* (Thunb.) (Sinomenii Caulis), *Polistes olivaceous* (DeGeer) (Vespae Nidus), and *Scolopendra subspinipes mutilans* L. Koch (Scolopendra) (**Table 1**). We have previously reported that compound QRHXD can reduce RA-related disease activity and attenuate the inflammatory response in clinical settings (11). Additionally, QRHXD may help prevent bone destruction in RA patients (12). Pharmacological studies have shown that Tanshinone IIA, the main active component of QRHXD, reduces disease severity and mitigates bone loss in an adjuvant-induced arthritis (AIA) rat model (13). However, elucidating the intrinsic mechanism of QRHXD remains challenging due to its complex composition. Multiomics analysis provides a promising approach to uncovering mechanisms of action and identifying drug efficacy targets in inflammatory immune-based diseases (14).

**Table 1:** Specific ingredients of QRHXD.

Authority names	Scientific name	Plant part	Weight ratio
Atractylodis Rhizoma	*Atractylodes chinensis*	Root	15
Phellodendri Chinensis Cortex	*Phellodendron amurense*	Tree bark	9
Smilacis glabrae rhizoma	*Smilax glabra* Roxb	Root	30
Lonicerae Japonicae Flos	*Lonicera japonica* Thunb	Bud	30
Astragali Radix	*Astragalus membranaceus* (Fisch.) Bge.	Root	30
Paeoniae Radix Rubra	*Paeonia lactiflora* Pall	Root	15
Dioscoreae Spongiosae Rhizoma	*Dioscorea futschauensis* Uline ex R. Kunth	Root	15
Salviae Miltiorrhizae Radix Et Rhizoma	*Salvia miltiorrhiza* Bge.	Root	15
Curcumae Rhizoma	*Curcuma phaeocaulis* Val.	Root	9
Sinomenii Caulis	*Sinomenium acutum* (Thunb.)	Stem	15
Vespae Nidus	*Polistes olivaceous* (DeGeer)	Nest	5
Scolopendra	*Scolopendra subspinipes mutilans* L. Koch	Body	4

Therefore, we conducted a 24-week clinical study in which QRHXD served as the primary treatment, collecting serum samples from patients before and after treatment with QRHXD for integrated proteomic and metabolomic analyses to identify potential therapeutic targets. Furthermore, a collagen-induced arthritis (CIA) mouse model was constructed to validate QRHXD’s efficacy and targets using Western blotting (WB), micro-CT, haematoxylin-eosin (HE), and other methods, with the goal of identifying new therapeutic methods for treating RA.

## Materials and methods

### Preparation of drugs and chemical reagents

QRHXD was prepared and supplied by Sichuan New Green Pharmaceutical Technology Development Co. Ltd., while MTX was supplied by Shanghai Xinyi Pharmaceutical Co. Ltd. Collagen-Type II bovine (batch number: 20022) and Complete Freund’s Adjuvant (batch number: 7009) were purchased from Beijing BioDee Biotechnology Co. Ltd. AMPK antibody (batch number: AF6423) and p-AMPK antibody (batch number: AF3423) were obtained from Affinity Biosciences and the actin antibody (batch number: GB15003) was purchased from Wuhan Servicebio Technology Co. Ltd. The HE kit (batch number: G1120) was acquired from Beijing Solarbio Science & Technology Co. Ltd. Additionally, the human fructose-1,6-bisphosphatase-1 (FBP1) antibody (batch number: A11664), FBP1 kit (batch number: RK10840), and multifactor kit (batch number: RK04321) were purchased from ABclonal Technology Co. Ltd.

### LC-MS/MS of QRHXD

The active ingredients of QRHXD were determined using liquid chromatography-mass spectrometry/mass spectrometry (LC-MS/MS). A total of 0.1 g of the prescribed extract was added to 20 mL of methanol and sonicated for 30 min until completely dissolved. Next, 1 mL of the extract was diluted to a volume of 10 mL, filtered through a 0.22-µm microporous filter membrane, and analysed using the Agilent 1260 Liquid Chromatography System, which includes two solvent delivery systems, a degasser, a column oven, an autosampler, and a workstation. The chromatographic column used was an Agilent Poroshell 120 SB C18 (100 mm × 2.1 mm, 2.7 μm), with a mobile phase A consisting of water containing 0.1% ammonium formate and mobile phase B being acetonitrile. The flow rate was set to 0.3 mL/min. The elution gradient was as follows: 0.00 min, 10% B; 0.50 min, 20% B; 7.50 min, 45% B; 8.50 min, 90% B; 12.00 min, 90% B; 12.10 min, 10% B; and 15.00 min, 10% B.

The MS data were collected using an Applied Biosystems API 4500 Qtrap liquid mass spectrometry system in MRM mode. An ESI source was used with simultaneous scanning of positive and negative ions. The MS parameters were as follows: ion source temperature, 500°C; ion source voltage, 5,500 V/− 4,500 V; atomising gas, N2; collision gas CAD, 6 psi; curtain gas CUR, 35 psi; atomising gas GS1, 50 psi; and auxiliary gas GS2, 50 psi. The declustering potential (DP) and collision energy (CE) were configured as listed in **Table 2**. The precursor and product ions for these compounds were set accordingly (**Table 2**).

**Table 2:** Results of LC-MS/MS analysis of QRHXD.

Name of compound	Retention time (min)	Result (mg/g)	Parent ion (m/z)	Daughter ion (m/z)	DP (V)	CE (eV)	CV (V)
Chlorogenic acid	2.95	11.3744	354.309	191.0^a^/160.7	− 55	− 30	40
Sinomenine	2.97	1.7900	329.396	207.0/181.0*	90	45	30
Paeoniflorin	3.57	2.8698	480.46	449.2*/327.0	− 65	− 15	40
Calycosin-7-*O*-glucoside	3.93	0.1597	446.4	285.1*/270.1	60	55	40
Astilbin	4.48	0.5185	450.396	285.0/151.1*	− 90	− 30	40
Atractylodin	5.21	0.0751	182.2179	127.2^a^/99.3	45	25	30
Berberine hydrochloride	7.23	0.2957	371.81	320.0*/292.0	80	35	40
Curcumin	10.51	0.0082	368.38	177.0*/145.0	60	45	40
Tanshinone IIA	11.68	0.0017	294.34	277.3*/249.2	90	25	30

*Quantitative ion.

### Participant recruitment

Previously, we conducted a multicentre, randomised, double-blinded, controlled trial lasting 24 weeks to evaluate the efficacy of QRHXD against RA (ClinicalTrials.gov ID: NCT04170504). A total of 204 patients were randomly assigned in a 1:1 ratio to receive either QRHXD or placebo, in addition to MTX as the basic treatment. As a single centre, Guang’anmen Hospital was responsible for recruiting 18 RA patients for each of the treatment and control groups between January 2019 and December 2021. In this study, we collected serum samples from 18 RA patients in the treatment group before and after enrolment for proteomics and metabolomics analyses. Additionally, 10 healthy individuals were recruited as the control group. All participants voluntarily signed informed consent forms. The study was approved by the ethics committee of Guang’anmen Hospital (No. 2019-073-KY-01).

### Inclusion and exclusion criteria

The inclusion criteria were as follows: (1) patients diagnosed with RA based on the 2010 American College of Rheumatology (ACR)/European League Against Rheumatism (EULAR) RA classification criteria (15); (2) age over 18 years; (3) DAS-28 score exceeding 3.2; (4) not taking NSAIDs in the last 4 weeks; and (5) not taking glucocorticosteroids in the last 4 weeks or taking oral hormone doses that had stabilised at less than 10 mg/day.

The exclusion criteria were as follows: (1) patients with serious diseases affecting other organs; (2) those with other autoimmune diseases, such as desiccation syndrome; (3) individuals who are pregnant, breastfeeding, or have a recent birth plan; (4) those allergic to MTX or *Sinomenium acutum* (Thunb.); (5) patients previously treated with DMARDs (except MTX) or biologics that were discontinued for less than 4 weeks; (6) patients previously treated with leflunomide (LEF) that was discontinued for less than 12 weeks; (7) any other condition that investigators deem unsuitable for inclusion in this clinical trial.

### Treatment and sample collection

The QRHXD granules were packaged in 10 g bags. Eighteen patients received QRHXD twice daily for 24 weeks (taken by brewing one bag with boiled water and consumed at 0.5 h after breakfast and dinner). This treatment was combined with a base medication of 10 mg of MTX taken once weekly. Blood samples were collected from all subjects in the morning after overnight fasting. Serum was obtained after centrifugation at 3,000 rpm for 15 min, then aliquoted into serum tubes and stored at − 80°C. Serum samples from 17 patients were collected, as one patient’s sample was deemed invalid.

### Untargeted metabolomic study

#### Sample preparation

Each serum sample (150 µL) was thawed at room temperature and transferred to a 1.5-mL Eppendorf tube. A mixture of 400 µL of methanol and 200 µL of acetonitrile was then added. The samples were vortexed for 1 min, subjected to ultrasonication for 10 min in an ice-water bath, and stored overnight at − 40°C. Following extraction, the samples were centrifuged at 4°C (12,000 rpm) for 10 min. A total of 150 µL of the supernatant was collected and filtered through a 0.22-µm organic phase pinhole filter into an LC injection vial for LC-MS analysis.

#### LC-MS/MS analysis

Metabolic profiling was conducted in both ESI-positive and ESI-negative ion modes using an ACQUITY UPLC I-Class plus coupled with a Waters ACQUITY UPLC I-Class plus/Thermo QE plus, which was equipped with a heated electrospray ionisation (ESI) source. The analysis was performed on ACQUITY UPLC HSS T3 column (100 mm × 2.1 mm, 1.8 µm) maintained at 45°C. The mobile phase consisted of A-water (containing 0.1% formic acid) and B-acetonitrile, with a flow rate of 0.35 mL/min and an injection volume of 3 µL. The elution gradient was as follows: 0 min, 5% B; 2 min, 5% B; 4 min, 30% B; 8 min, 50% B; 10 min, 80% B; 14 min, 100% B; 15 min, 100% B; 15.1 min, 5% B; and 16 min, 5% B. The mass range was set from 100 to 1,200 m/z, with a primary MS scan resolution of 70,000, a secondary MS scan resolution of 17,500, and collision energies of 10, 20, and 40 eV, respectively. The mass spectrometer was operated according to normal standards.

#### Data preprocessing and statistical analysis

The original data were baseline filtered, peak identified, integrated, retention time corrected, peak aligned, and normalised using Progenesis QI V2.3 software. Compound characterisation was performed using the Human Metabolome Database (HMDB), Lipidmaps (V2.3), Metlin, and self-built databases, based on accurate mass-charge ratios (M/z), secondary fragmentation, and isotopic distributions. After characterising the compounds, the positive and negative ion data were combined and imported into the R package for principal component analysis (PCA) to assess the overall distribution and stability among samples. Group differences were analysed using orthogonal partial least squares discriminant analysis (OPLS-DA) and partial least squares discriminant analysis (PLS-DA). A *t*-test was conducted to determine the statistical significance of these differences, and differential metabolites (DMs) were identified based on VIP values greater than 1.0 and *p*-values less than 0.05.

### DIA proteomic study

#### Nanomagnetic bead-mediated enrichment of low-abundance plasma proteins

Low-abundance proteins in the serum samples were enriched using the EasyPept DeeP Kit for protein enrichment and pretreatment (Omicron, Shanghai, China). Following the manufacturer’s protocol, 1 mg (40 µL) of magnetic nanoparticle suspension was utilised, and the magnetic beads were isolated via magnetic separation to remove the supernatant. After several washes, the magnetic beads were resuspended, and 100 µL of serum was added. The mixture was then incubated at 37°C for 1 h with 360° rotation using a flip mixer. The supernatant was subsequently separated via magnetic separation, followed by the addition of 300 µL of washing solution and gentle shaking for 5 min. This washing step was repeated three times. The proteins were enzymatically hydrolysed to generate peptides, followed by reductive alkylation and desalting. The peptide concentration of each sample was determined using a Nanodrop.

#### Data-independent acquisition mass spectrometry analysis

All analyses were conducted using a TimsTOF Pro mass spectrometer equipped with an EASY-nLCTM 1200 system. The data-independent acquisition (DIA) liquid phase elution gradient was as follows: 0 min, 5% B; 45 min, 27% B; 50 min, 46% B; 55 min, 100% B; and 60 min, 100% B. The DIA mass spectrometry scanning parameters included a capillary voltage of 1.4 kV, a dry temperature of 180°C, a drying gas rate of 3.0 L/min, a mass range of 100–1,700 m/z, an ion mobility between 0.7 and 1.3 Vs/cm², and a collision energy range of 20–59 eV.

#### Database search and statistical analyses

The DIA original data were processed using Spectronaut Pulsar 17.5 software, with parameter settings and analysis conducted according to the user manual. Differentially expressed proteins (DEPs) were identified based on thresholds of a fold change > 2 or < 0.5 and a *p*-value < 0.05. Furthermore, the Kyoto Encyclopedia of Genes and Genomes (KEGG) and Gene Ontology (GO) enrichment analyses were performed to annotate the functions and pathways of the DEPs. Interaction analysis between DEPs was performed using String (https://string-db.org/).

### Animal experiments

#### Animals

Forty female DBA mice (6–8 weeks old, 18 g ± 22 g) were obtained from Beijing Vital River Laboratory Animal Technology Co. Ltd. (Licence No. SYXK [Beijing] 2023-0011). The mice were housed in an SPF-grade animal facility at a constant temperature of 24°C, under a 12-h light/dark cycle, with free access to food and water. The experimental mice were first adaptively fed for 1 week, after which the animals were subjected to modelling, pharmacological interventions, and specimen sampling in accordance with the principles of animal welfare. This experiment was approved by the ethics committee of Guang’anmen Hospital, China Academy of Chinese Medical Sciences (No. IACUC-GAMH-2019-001).

#### Preparation of drugs and reagents

The QRHXD solution for mice was prepared by dissolving QRHXD granules in hot water to obtain medicinal juice at concentrations of 1.35, 2.7, and 5.4 g/mL, which was then stored at − 20°C in the dark. The MTX solution was prepared by dissolving 5 mg of MTX in 84 mL of saline, to achieve a final concentration of 0.06 mg/mL, and the mixture was stored at − 20°C in the dark. For collagen preparation, bovine type II collagen and complete Fuchs’ adjuvant were mixed thoroughly at a 1:1 ratio on ice using a three-way tube until fully emulsified, resulting in an antigen concentration of 2 mg/mL.

#### CIA model establishment and treatment

The mice were initially immunised by subcutaneous injection of 0.2 mL of antigen into the root of the tail after 1 week of adaptive feeding. A booster injection of 0.2 mL of antigen was administered subcutaneously at the same site on day 21 days. The appearance of redness and swelling in the foot and paw after day 21 indicated successful modelling.

After successful modelling, 40 mice were randomly divided into the normal group, model group, MTX group (1.2 mg/kg/week), QRHXD low-dose group (QRHXD-L, 13.5 g/kg/day), QRHXD medium-dose group (QRHXD-M, 27 g/kg/day), QRHXD high-dose group (QRHXD-H, 54 g/kg/day), with 8 mice in each group. The normal group and model group were given 0.2 mL of saline by gavage, once per day; the QRHXD group was given 0.2 mL of QRHXD solution by gavage, once per day; the MTX group was given 0.2 mL of MTX solution by gavage, twice per week.

Four weeks after administration, the mice were anaesthetised, and blood was collected from the eyeballs in centrifuge tubes. Then, the blood was centrifuged at 3,000 rpm for 15 min, and the supernatant was collected in a clean centrifuge tube and frozen at − 80°C for the inflammatory cytokine assay. Both the hind feet, liver, and kidney were removed from a clean environment. The right foot was frozen at − 80°C for WB detection; the left foot was placed in a paraformaldehyde tube for histopathological testing and micro-CT testing.

#### Toe swelling measurement and the arthritis index

Starting from the initial immunisation, the ankle joint leg diameters of both hind limbs of the mice were measured and averaged every 7 days using Vernier callipers. Additionally, joint swelling was assessed twice a week for arthritis index (AI) scoring. The AI scoring criteria were as follows: on a scale of 0–4, no inflamed joints were scored as 0, the presence of inflamed toe joints was scored as 1, both toe joints and inflamed metatarsals were scored as 2, joints below the inflamed ankle were scored as 3, and whole-foot inflamed joints, including the ankle joints, were scored as 4. The scores of the mouse limbs were recorded and summed each time, with a maximum cumulative score of 16 points.

#### Histopathology

The left hindfoot, liver, and kidney of each mouse were fixed in universal tissue fixative for 48 h and then decalcified for 6 weeks. After complete decalcification, the tissues were paraffin-embedded, sectioned, HE-stained, observed under a light microscope, and photographed.

#### Micro-CT and bone indices

The left hind feet of the mice were removed from the tissue fixative, positioned using a polystyrene tube to ensure alignment with the centre of rotation, and placed in a micro-CT machine. The region of interest (ROI) in the camera’s field of view was located, the pixel size was set, the filter was corrected, the voltage was adjusted, and scanning begins was initiated. Subsequently, 3D image reconstruction and analysis were performed. The bone surface area (BS), bone volume (BV), BS/BV, number of trabeculae (Tb.N), thickness of the trabeculae (Tb.Th), and degree of separation of the trabeculae (Tb.Sp) were analysed across different groups of mice.

#### Inflammatory cytokine assay

Mouse serum was removed from a − 80°C refrigerator, incubated at room temperature, and processed according to the kit instructions to detect interleukin (IL)-1β, IL-6, IL-10, IL-17, and tumour necrosis factor α (TNF-α) levels.

#### Western blot

The synovial tissues of the right hind foot of each mouse were collected, dissociated, and lysed with RIPA buffer. Protein concentration was determined simultaneously. SDS-PAGE gels (10%) were used for protein electrophoresis, and the separated proteins were transferred to PVDF membranes and blocked with 5% nonfat milk at room temperature. The target proteins were detected using primary antibodies. After being washed three times with TBST, the membranes were incubated with secondary antibodies. β-Actin served as an internal reference.

### Statistical methods

The data are presented as means ± SDs and were analysed using GraphPad Prism 10.0. One-way ANOVA was performed to compare different groups. A *p*-value of < 0.05 was considered statistically significant.

## Results

### Identification of the constituents of QRHXD via LC-MS/MS

**Figure 1:**
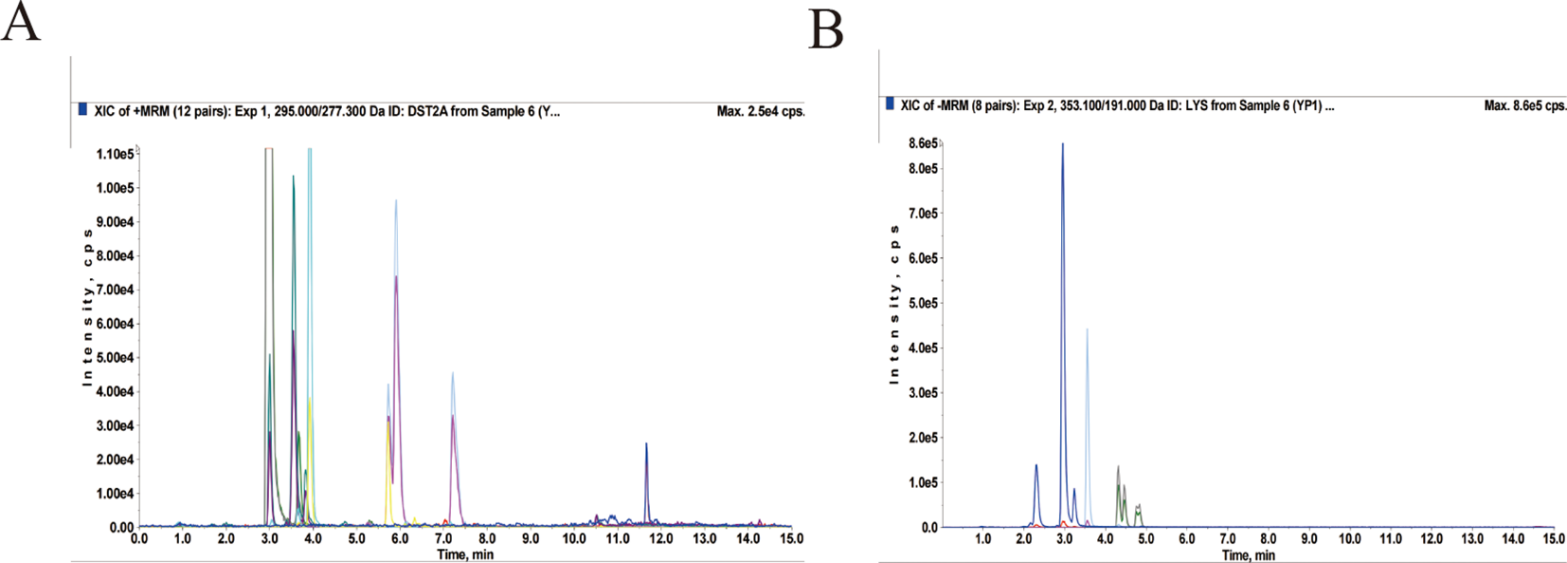
Total ion chromatogram of QRHXD in positive ion mode **(A)** and negative ion mode **(B)** of LC-MS/MS.

LC/MS-MS was utilised to establish the quality control of QRHXD and identify its compounds. Clear chromatographic separation and strong mass spectral responses were achieved for each target compound using the optimised LC-MS/MS conditions. For example, chlorogenic acid had a retention time of 2.95 min and a quantitative signal-to-noise ratio exceeding 10. By comparing QRHXD chromatograms with standards, nine active ingredients were identified: chlorogenic acid, sinomenine, paeoniflorin, calycosin-7-*O*-glucoside, astilbin, atractylodin, berberine hydrochloride, curcumin, and tanshinone IIA. Full details are presented in **Table 2**; **Figure 1**.

### Baseline data characteristics and comparisons of clinical efficacy indicators

As shown in **Table 3**, there was no significant difference (*p* > 0.05) among the LQ group (baseline), LH group (patients after 24 weeks of treatment), and ZC group (healthy adults) in terms of age, sex, height, weight, temperature (T), pulse (P), respiration (R), systolic blood pressure (SBP), diastolic blood pressure (DBP), white blood cell (WBC), red blood cell (RBC), haemoglobin (HGB), aspartate aminotransferase (AST), alanine aminotransferase (ALT), platelet (PLT), blood urea nitrogen (BUN), and creatinine (Cr) levels. These findings indirectly indicate that QRHXD has satisfactory safety. As shown in **Table 4**, significant differences (*p* < 0.05) were observed between the LQ and LH groups in the primary outcome, disease activity score-28 (DAS28), as well as in the secondary outcomes, including the visual analogue scale (VAS) score, C-reactive protein (CRP) level, erythrocyte sedimentation rate (ESR), tender joint count (TJC), swollen joint count (SJC), patient-reported outcome (PRO), and health assessment questionnaire (HAQ). However, no significant differences (*p* > 0.05) were found in the rheumatoid factor (RF) score. These findings suggest that QRHXD can reduce RA disease activity, alleviate clinical symptoms, and mitigate the inflammatory response.

**Table 3:** Basic information characteristics of subjects.

Variables	Baseline	24 weeks	Healthy adults	*p*-value
	LQ group (*n* = 17)	LH group (*n* = 17)	ZC group (*n* = 10)	
Age (years)	46.29 ± 11.49	46.29 ± 11.49	40.80 ± 7.04	0.368
Female (%)	14 (82.35)	14 (82.35)	6 (60)	0.365
Height (cm)	163.06 ± 3.93	163.06 ± 3.93	165.60 ± 7.55	0.371
Weight (kg)	55.71 ± 4.27	56.03 ± 4.27	57.90 ± 9.17	0.594
SBP (mmHg)	118.82 ± 4.64	117.88 ± 4.12	117.10 ± 2.92	0.564
DBP (mmHg)	78.53 ± 3.47	78.71 ± 3.64	78.90 ± 3.21	0.964
R	17.76 ± 1.68	17.53 ± 1.23	17.00 ± 1.33	0.418
P	75.35 ± 6.15	75.24 ± 5.72	75.50 ± 5.89	0.994
T (°C)	36.47 ± 0.22	36.41 ± 0.17	36.34 ± 0.33	0.375
WBC	6.57 ± 1.75	6.06 ± 1.72	5.54 ± 1.23	0.291
RBC	4.40 ± 0.43	4.28 ± 0.41	4.13 ± 0.48	0.309
HGB	128.18 ± 15.84	130.12 ± 11.95	125.60 ± 13.44	0.718
PLT	252.59 ± 52.08	252.00 ± 49.47	231.60 ± 48.15	0.524
ALT	15.14 ± 6.34	16.22 ± 5.26	15.81 ± 4.49	0.850
AST	19.49 ± 5.55	19.21 ± 5.96	21.80 ± 6.48	0.516
BUN	4.33 ± 1.03	4.23 ± 1.23	4.24 ± 1.11	0.957
Cr	51.53 ± 4.91	49.88 ± 10.29	50.50 ± 5.46	0.816

*LQ group*, RA patients before treatment with QRHXD; *LH group*, RA patients after treatment with QRHXD; *ZC group*, healthy adults; *SBP*, systolic blood pressure; *DBP*, diastolic blood pressure; *R*, respiration; *P*, pulse; *T*, temperature; *WBC*, white blood cell; *RBC*, red blood cell; *HGB*, haemoglobin; *PLT*, platelet; *ALT*, alanine aminotransferase; *AST*, aspartate aminotransferase; *BUN*, blood urea nitrogen; *Cr*, creatinine.

**Table 4:** Comparison of clinical indicators before and after treatment with QRHXD.

Outcome measures	Baseline	24 weeks	*p*-value
	LQ group (*n* = 17)	LH group (*n* = 17)	
Primary outcome			
DAS28	4.78 ± 0.97	2.76 ± 0.98	0.000
Secondary outcome			
VAS	6.06 ± 1.20	3.06 ± 1.52	0.000
TJC	6.35 ± 3.90	2.53 ± 2.53	0.002
SJC	4.06 ± 3.01	0.82 ± 1.47	0.000
ESR	28.94 ± 19.64	15.00 ± 11.96	0.018
CRP	16.27 ± 20.52	3.95 ± 4.55	0.027
HAQ	1.04 ± 0.70	0.29 ± 0.35	0.001
PRO	7.53 ± 2.29	3.75 ± 2.39	0.000
RF	225.94 ± 284.57	118.94 ± 143.38	0.176

*LQ group*, RA patients before treatment with QRHXD; *LH group*, RA patients after treatment with QRHXD; *DAS28*, Disease Activity Score-28; *VAS*, visual analogue scale; *TJC*, tender joint count; *SJC*, swollen joint count; *ESR*, erythrocyte sedimentation rate; *CRP*, C-reactive protein; *HAQ*, health assessment questionnaire; *PRO*, patient-reported outcome; *RF*, rheumatoid factor.

### Proteomic analysis

#### DEPs among the three groups

**Figure 2:**
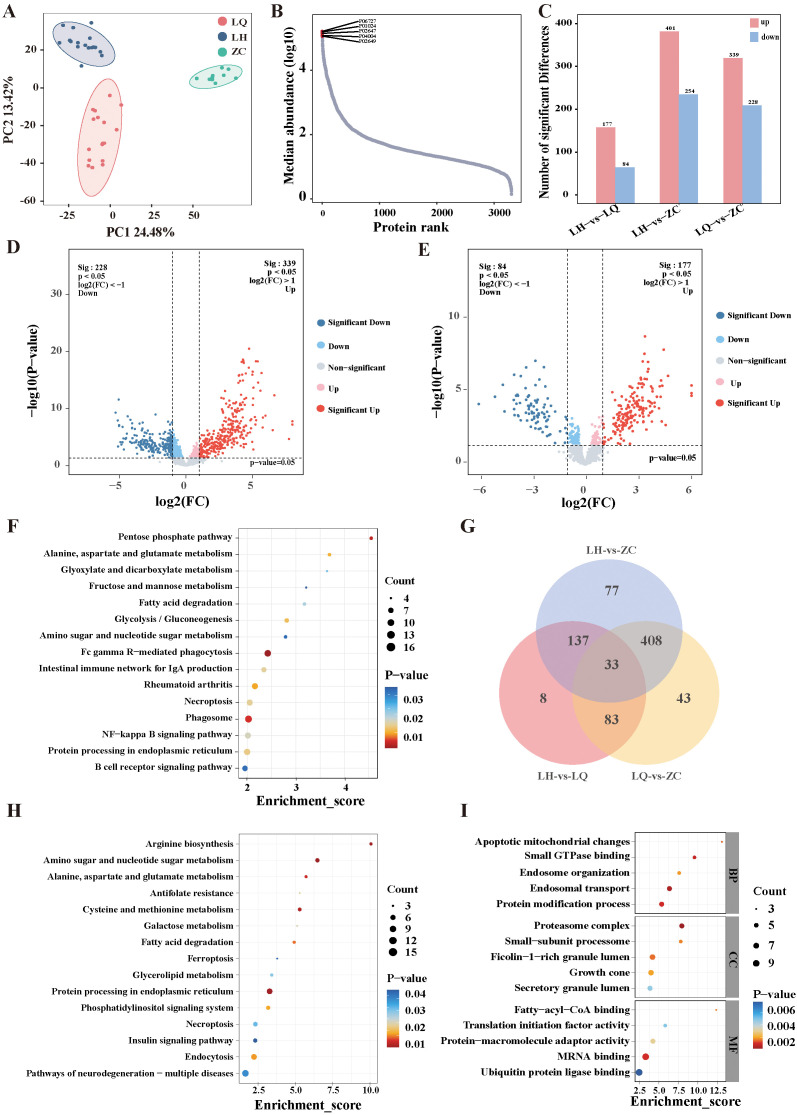
Proteomic analysis of RA patients before treatment with QRHXD (LQ Group), RA patients after treatment with QRHXD (LH Group) and healthy subjects (ZC Group). **(A)** Principal component analysis (PCA) of differentially expressed proteins (DEPs). **(B)** Relative abundance of proteins spans five orders of magnitude. **(C)** DEPs statistics for the 3 groups. **(D)** DEPs volcano plots of the LQ Group and ZC Group. **(E)** DEPs volcano plots of the LH Group and LQ Group. **(F)** KEGG enrichment analysis of DEPs between LQ Group and ZC Group. **(G)** Venn intersection analysis of DEPs in 3 groups. (H) KEGG enrichment analysis of DEPs between LH Group and LQ Group. **(I)** GO enrichment analysis of DEPs between LH Group and LQ Group.

We analysed the collected serum samples via DIA proteomics. PCA (**Figure 2A**) revealed that the samples were well aggregated within the same group, with pronounced differences between groups. A total of 3,305 proteins were identified in this study, with their relative abundance spanning five orders of magnitude. The highest protein level was observed for apolipoprotein A4 (APOA4), while the lowest was for apolipoprotein E (APOE) (**Figure 2B**). **Figures 2C–E** show 177 upregulated and 84 downregulated proteins in the LH group compared with the LQ group, 339 upregulated and 228 downregulated proteins in the LQ group compared with the ZC group, and 401 upregulated and 254 downregulated proteins in the LH group compared with the ZC group. We then performed Venn intersection analysis on the DEPs and found that 83 proteins differed in both the LH and LQ groups, while there was no difference in the LH and ZC groups, suggesting that these proteins may be therapeutic targets of QRHXD (**Figure 2G**).

#### GO and KEGG enrichment analyses of DEPs

The functions and biological pathways of the DEPs were analysed using KEGG and GO pathway analyses. KEGG pathway analysis revealed that pathogenic proteins associated with RA were primarily involved in the B-cell receptor signalling pathway; protein processing in the endoplasmic reticulum; NF-kappa B signalling pathway; phagosome; necroptosis; rheumatoid arthritis; intestinal immune network for IgA production; Fc gamma R-mediated phagocytosis; amino sugar and nucleotide sugar metabolism; glycolysis/gluconeogenesis; fatty acid degradation; fructose and mannose metabolism; glyoxylate and dicarboxylate metabolism; alanine, aspartate, and glutamate metabolism; and the pentose phosphate pathway (**Figure 2F**). The therapeutic proteins of QRHXD were involved principally in pathways of neurodegeneration—multiple diseases; endocytosis; insulin signalling pathway; necroptosis; phosphatidylinositol signalling system; protein processing in the endoplasmic reticulum; glycerolipid metabolism; ferroptosis; fatty acid degradation; galactose metabolism; cysteine and methionine metabolism; antifolate resistance; alanine, aspartate, and glutamate metabolism; amino sugar and nucleotide sugar metabolism; and arginine biosynthesis (**Figure 2H**). GO analysis revealed that the DEPs between the LH and LQ groups were associated mainly with protein modification process, endosomal transport, endosome organisation, small GTPase binding, apoptotic mitochondrial changes, growth cone, ficolin-1-rich granule lumen, small-subunit processome, proteasome complex, ubiquitin protein ligase binding, MRNA binding, protein-macromolecule adaptor activity, translation initiation factor activity, and fatty-acyl-CoA binding (**Figure 2I**).

### Metabolomics analysis

#### DMs between the three groups

**Figure 3:**
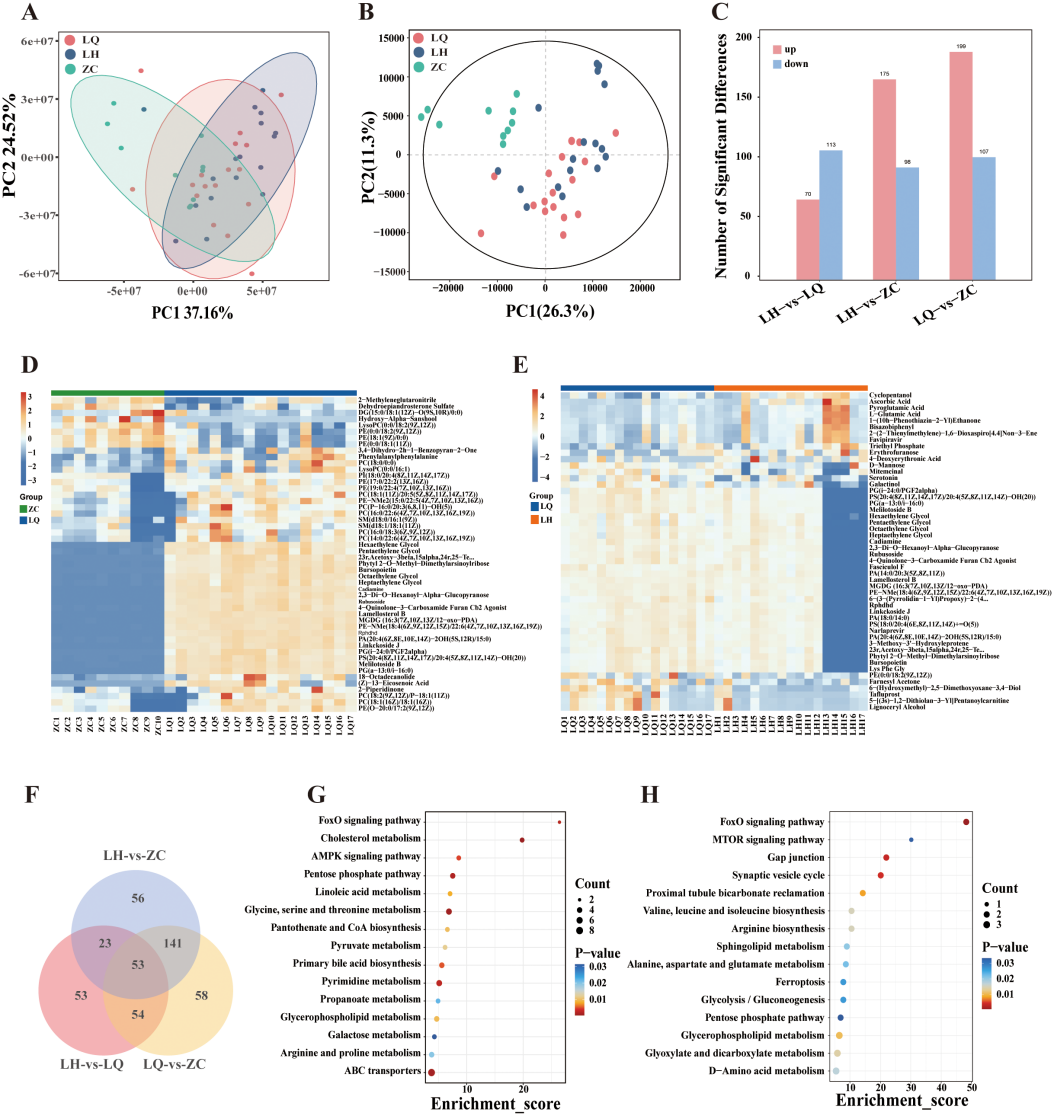
Metabolomics analysis of RA patients before treatment with QRHXD (LQ Group), RA patients after treatment with QRHXD (LH Group) and healthy subjects (ZC Group). **(A)** Principal component analysis (PCA) of differential metabolites (DMs). **(B)** Partial Least Squares Discriminant Analysis (PLS-DA) of DMs. **(C)** DMs statistics for the 3 groups. **(D)** Cluster heatmap of DMs between LQ Group and ZC Group. **(E)** Cluster heatmap of DMs between LH Group and LQ Group. **(F)** Venn intersection analysis of DMs between 3 groups. **(G)** KEGG enrichment analysis of DMs between LQ Group and ZC Group. **(H)** KEGG enrichment analysis of DMs between LH Group and LQ Group..

The PCA model diagram indicated that the QC samples were closely clustered, demonstrating satisfactory detection stability (**Figure 3A**). DMs between the groups were distinguished using partial least squares discriminant analysis (PLS-DA), which revealed significant differences among groups (**Figure 3B**). The metabolomics analysis identified 70 upregulated and 133 downregulated metabolites in the LH group compared with the LQ group, 199 upregulated and 107 downregulated metabolites in the LQ group compared with the ZC group, and 175 upregulated and 98 downregulated metabolites in the LQ group compared with the ZC group (**Figures 3C–E**). A subsequent Venn intersection analysis of the DMs identified 54 metabolites that differed in both the LH and LQ groups, with no differences observed between the LH and ZC groups, suggesting that these metabolites may serve as therapeutic targets of QRHXD (**Figure 3F**).

#### KEGG enrichment analyses of DMs

KEGG enrichment analyses of DMs clearly demonstrated that metabolic processes are closely associated with statistically significant pathway maps. The top 15 statistically significant pathways of the pathogenic DMs are presented in **Figure 3G** and include ABC transporters; arginine and proline metabolism; galactose metabolism; glycerophospholipid metabolism; propanoate metabolism; pyrimidine metabolism; primary bile acid biosynthesis; pyruvate metabolism; pantothenate and CoA biosynthesis; glycine, serine, and threonine metabolism; linoleic acid metabolism; pentose phosphate pathway; AMPK signalling pathway; cholesterol metabolism; and FoxO signalling pathway. The therapeutic metabolites of QRHXD were principally involved in d-amino acid metabolism; glyoxylate, and dicarboxylate metabolism; glycerophospholipid metabolism; the pentose phosphate pathway; glycolysis/gluconeogenesis; ferroptosis, alanine, aspartate, and glutamate metabolism; sphingolipid metabolism; arginine biosynthesis; valine, leucine, and isoleucine biosynthesis; proximal tubule bicarbonate reclamation; the synaptic vesicle cycle; gap junctions; the MTOR signalling pathway; and the FoxO signalling pathway (**Figure 3H**).

### Integrated analysis of DEPs and DMs

**Figure 4:**
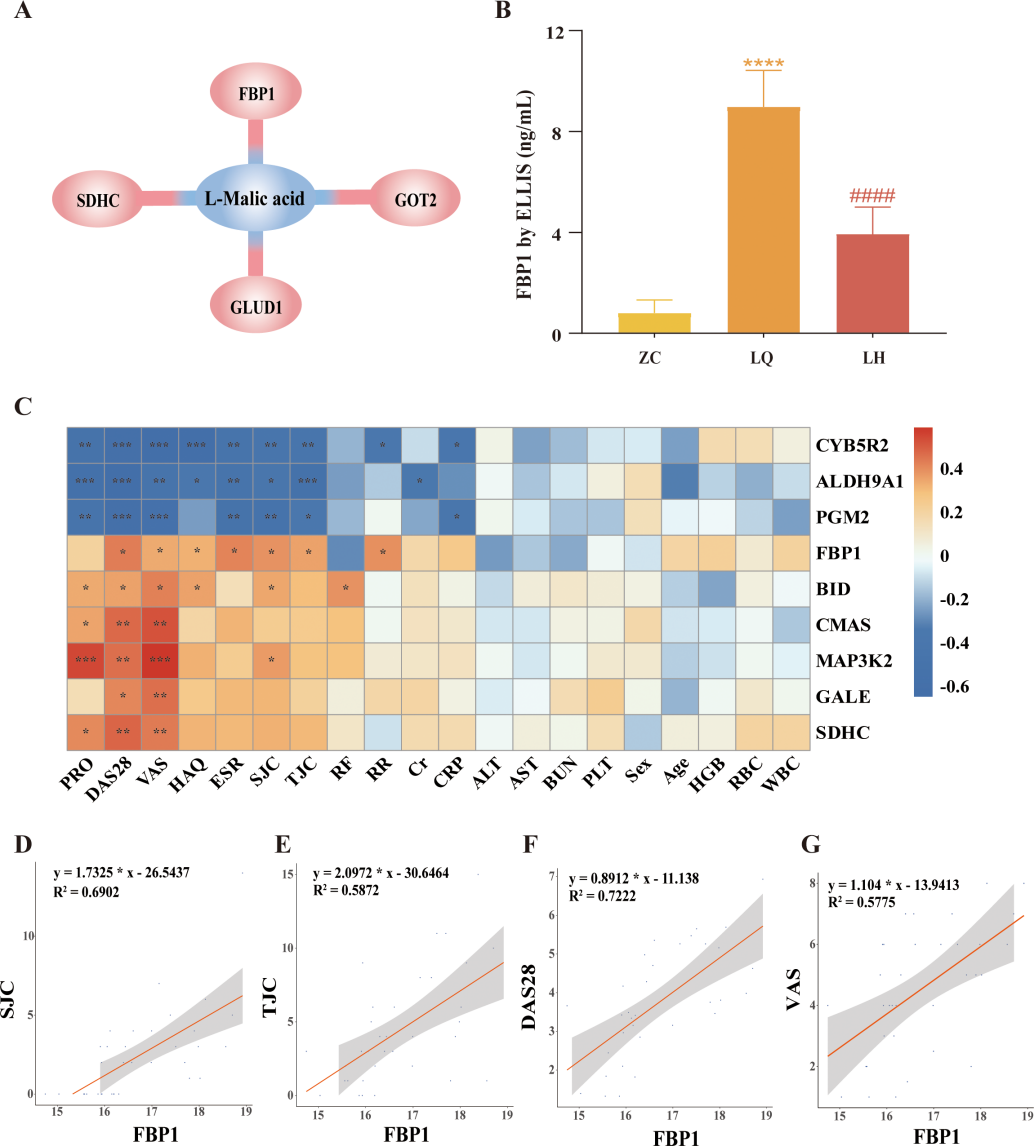
Integrated analysis of differentially expressed proteins (DEPs) and differential metabolites (DMs). **(A)** L-Malic acid and FBP1 are biologically related. **(B)** Validation of FBP1 in human serum by ELISA. ****p<0.0001, compared with ZC Group. ^####^ *p*<0.0001, compared with LQ Group. **(C)** Heatmap of clustering of 9 DEPs with clinical indicators. *p<0.05, **p<0.01, ***p<0.001. **(D)** Spearman analysis of FBP1 and swollen joint count (SJC). **(E)** Spearman analysis of FBP1 and tender joint count (TJC). **(F)** Spearman analysis of FBP1 and the disease activity score-28 (DAS-28). **(G)** Spearman analysis of FBP1 and visual analogue scale (VAS).

A total of 83 DEPs and 54 DMs were entered into MetaboAnalyst (https://www.metaboanalyst.ca/), and the results revealed a biological between L-malic acid and FBP1 (**Figure 4A**). In addition, KEGG enrichment analysis of the 83 DEPs and 54 DMs revealed that proteins and metabolites with similar biological relationships clustered into several pathways, including the glucagon signalling pathway, AMPK signalling pathway, glycolysis/gluconeogenesis, galactose metabolism, amino sugar and nucleotide sugar metabolism, insulin signalling pathway, diabetic cardiomyopathy, pentose phosphate pathway, nonalcoholic fatty liver disease, and starch and sucrose metabolism (**Table 5**). Since FBP1 is a key protein in the association analysis and plays a crucial role in the AMPK pathway, we considered FBP1 and AMPK to be central to the pathogenesis of RA and potential therapeutic targets of QRHXD. To further verify this, we examined FBP1 and AMPK in CIA mice.

**Table 5:** Integrated analysis of differentially expressed proteins and differential metabolites.

Pathway	Protein
Glucagon signalling pathway	FBP1
Galactose metabolism	GALE; PGM2
AMPK signalling pathway	FBP1
Amino sugar and nucleotide sugar metabolism	GALE; CYB5R2; PGM2; CMAS
Insulin signalling pathway	FBP1
Diabetic cardiomyopathy	SDHC
Pentose phosphate pathway	FBP1; PGM2
Nonalcoholic fatty liver disease	BID; SDHC; MAP3K2
Glycolysis/gluconeogenesis	FBP1; PGM2; ALDH9A1
Starch and sucrose metabolism	PGM2

### Validation of candidate proteins and Spearman’s analysis of clinical indicators

Furthermore, we validated FBP1 levels in human serum samples using ELISA. The results showed that FBP1 was significantly higher in the LQ group compared to the ZC group and significantly lower in the LH group compared to the LQ group (**Figure 4B**). These findings were consistent with the multiomics analysis described earlier. Moreover, we performed Spearman’s correlation analysis between the target protein FBP1 and clinical indicators of RA patients. As shown in **Figures 4C–G**, elevated FBP1 levels were significantly associated with increased DAS-28, VAS, TJC, and SJC in RA patients. These results suggest that increased FBP1 may be a key factor contributing to high disease activity in RA. At the same time, QRHXD treatment was able to inhibit FBP1 expression in RA patients, potentially reducing disease activity.

### QRHXD inhibits synovial proliferation and arthritis in CIA mice

**Figure 5:**
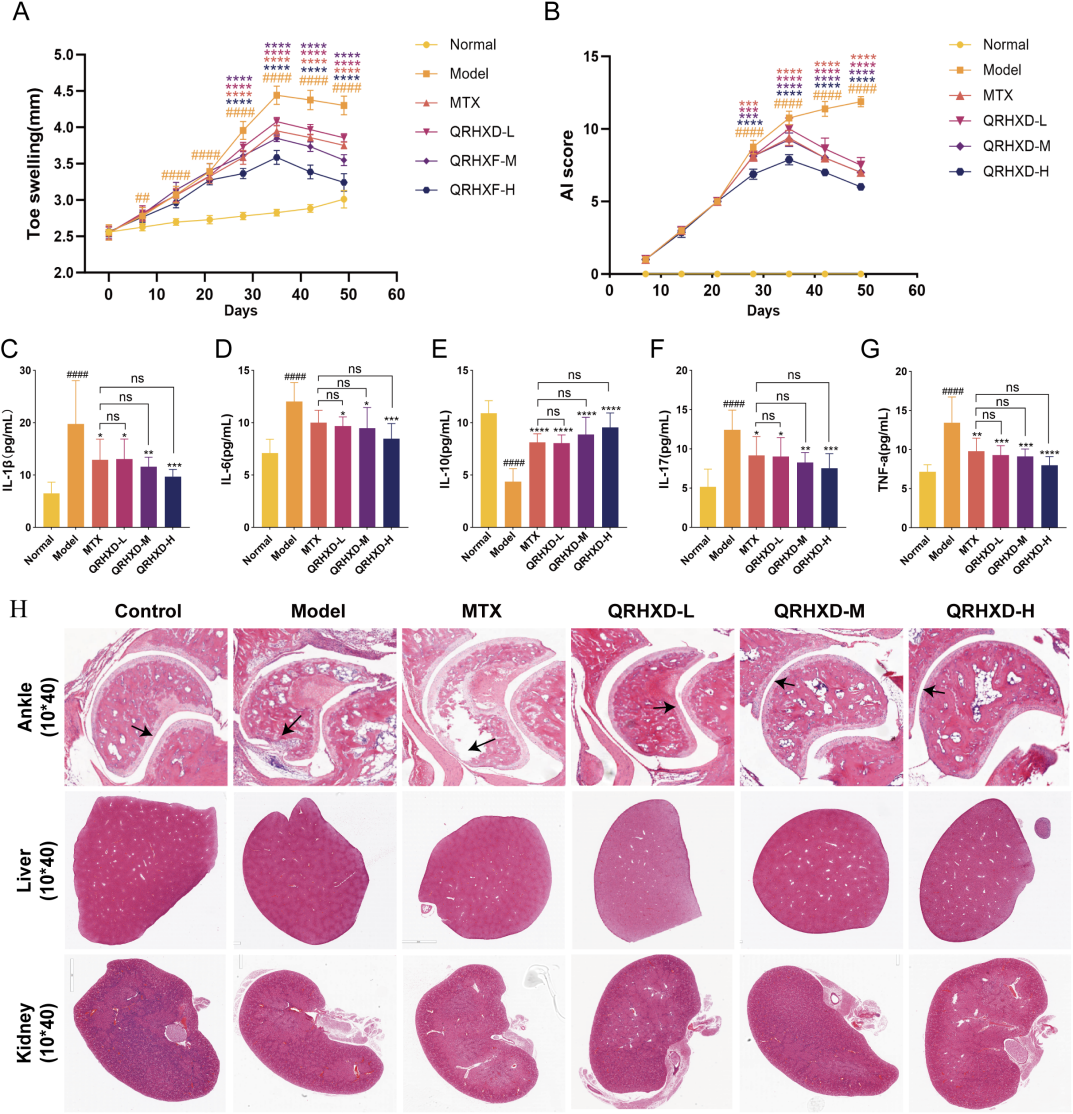
Effect of QRHXD on arthritis, toe swelling, synovial proliferation and inflammatory cytokines in CIA mice. **(A)** The degree of swelling of the toes. **(B)** Arthritis Index. **(C–G)** IL-10, IL-1β, IL-6, IL-17, and TNF-α in serum of mice. **(H)** HE of the ankle, liver and kidney in mice. ^##^ *p*<0.05, ^####^ *p*<0.0001, compared with Normal Group. *p<0.05, **p<0.01, ***p<0.001, ****p<0.0001, compared with Model Group. ^ns^ *p*>0.05, compared with MTX Group.

To evaluate the efficacy and mechanism of QRHXD, we constructed animal experiments using CIA model mice. CIA model mice developed more severe arthritis than normal control mice. QRHXD and MTX significantly reduced toe swelling and arthritis (**Figures 5A, B**), decreased inflammatory factors such as IL-1β, IL-6, IL-17, and TNF-α, and increased the anti-inflammatory factor IL-10 (**Figures 5C–G**). Additionally, QRHXD-H demonstrated superior efficacy compared to MTX, QRHXD-M, and QRHXD-L. Moreover, HE staining of the ankle joints revealed that, in the model group, the joint space narrowed, the articular surface was less smooth or even collapsed, and numerous infiltrating inflammatory cells and vascular opacification were observed. Compared with those in the model group, synovial hyperplasia, and joint stenosis were less severe in the QRHXD and MTX groups, with QRHXD-H showing the most pronounced improvement. Notably, HE staining of the livers and kidneys of CIA mice indicated that QRHXD has favourable safety (**Figure 5H**).

### QRHXD delays bone destruction through the FBP1/AMPK signalling pathway in CIA mice

**Figure 6:**
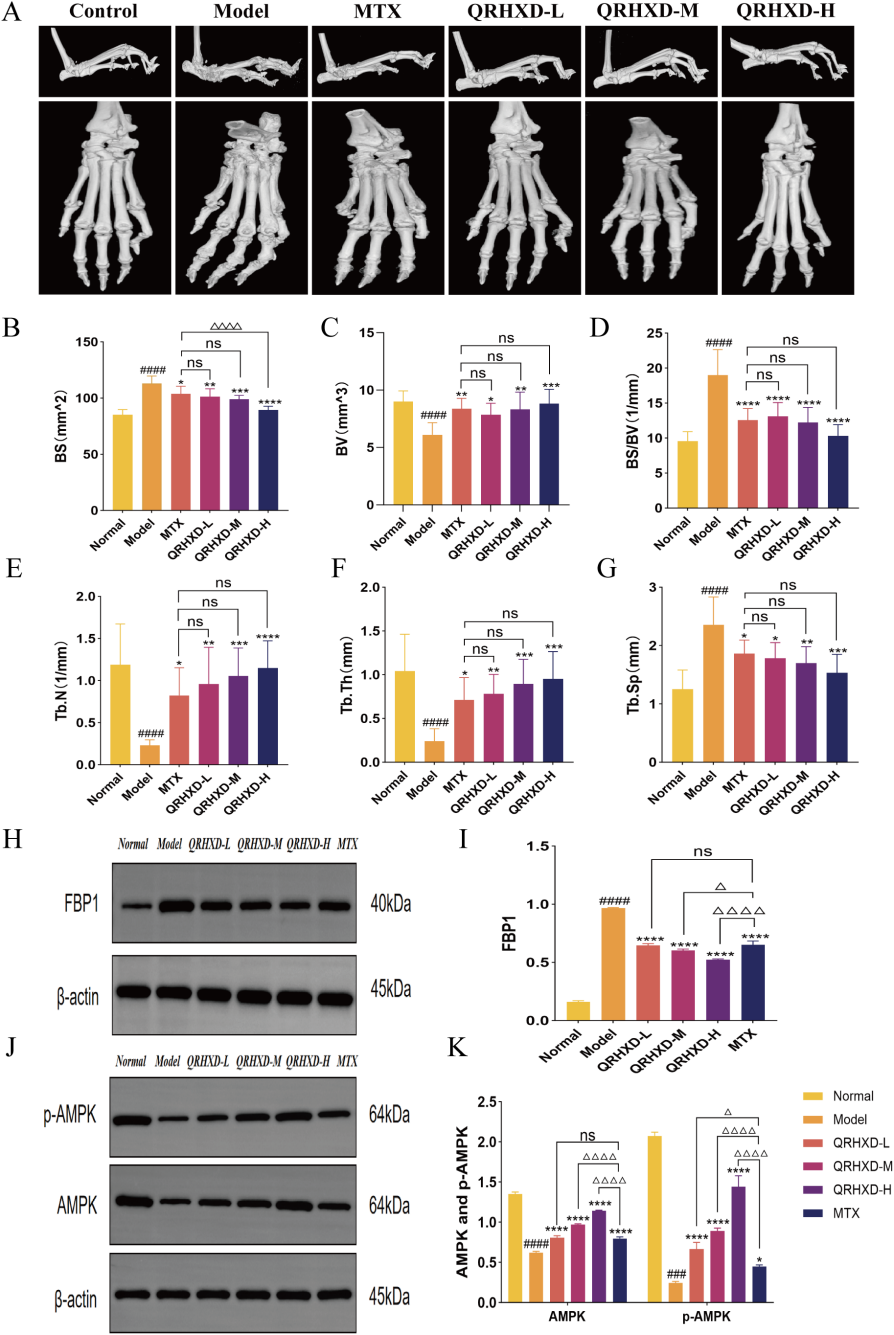
Assessment of bone destruction in mice and WB validation of target proteins. **(A)** Micro-CT scan of mice ankle joints. **(B–G)** The indexes of BV, BS, BS/BV, Tb.N, Tb.Th, and Tb.Sp in mice ankle joints. **(H, I)** Proteins express of FBP1. **(J, K)** Proteins express of AMPK and p-AMPK. ^####^ *p*<0.0001, compared with Normal Group. *p<0.05, **p<0.01, ***p<0.001, ****p<0.0001, compared with Model Group. ^ns^ *p*>0.05, compared with MTX Group. ^△^ *p*<0.05, ^△△△△^ *p*<0.0001, compared with MTX Group.

In addition, we assessed the protective effect of QRHXD against bone destruction using micro-CT scanning of the ankle joints in CIA mice and by collecting bone indices, including BV, BS, BS/BV, Tb.N, Tb.Th, and Tb.Sp. The results revealed that mice in the model group exhibited rough articular surfaces, joint deformation, and severe bone destruction. In this group, BS, BS/BV, and Tb.Sp were elevated, while BV, Tb.N, and Tb.Th were decreased. QRHXD reversed these effects and reduced bone destruction, with QRHXD-H demonstrating greater efficacy (**Figures 6A–G**). To confirm the effect of QRHXD on the FBP1/AMPK signalling pathway, we performed a WB analysis of mouse synovial membranes. The results revealed that FBP1 was elevated, while AMPK and p-AMPK were reduced in the model group. In addition, FBP1 levels decreased, and AMPK and p-AMPK levels increased in the QRHXD and MTX groups. Moreover, these proteins were significantly elevated in the QRHXD-H group (**Figures 6H–K**).

## Discussion

RA is an autoimmune disease characterised by progressive, irreversible joint damage, which can lead to permanent disability (16). Its prevalence and the number of affected people with disabilities continue to rise each year (17). Lifelong treatment is often necessary, and there is a consensus that early intervention and up-to-date therapeutic approaches are essential for controlling disease activity and delaying functional decline (18). The treatment goal is to reduce disease activity by at least 50% within 3 months and achieve remission or low disease activity status within 6 months (19). Currently, RA is primarily treated with DMARDs such as MTX, as well as biologics, including TNF-α inhibitors, IL-6 inhibitors, and JAK inhibitors. Despite advancements in disease management strategies and newer medications, a significant proportion of patients still experience uncontrolled disease (20). Additionally, the adverse effects of these treatments pose challenges for clinicians. For example, MTX can lead to elevated liver enzymes, pneumonia, and gastrointestinal discomfort; TNF inhibitors have been associated with demyelinating lesions; and JAK inhibitors may increase the risk of herpes zoster (21).

CM, with its long history, remains a treatment approach worth exploring. As an herbal compound, QRHXD has demonstrated a good efficacy against RA and a favourable safety profile. In this study, 17 RA patients showed improvements in DAS-28, HAQ, PRO, VAS, TJC, SJC, ESR, CRPF, and other clinical indicators after 24 weeks of QRHXD treatment, indicating that the formula effectively reduced RA disease activity, alleviated inflammation, and regulated somatic function. In addition, QRHXD helped delay RA-induced bone destruction. In CIA model mice, QRHXD intervention led to improvements in micro-CT results, bone indexes, histological evaluation (HE), and inflammatory factor levels, with the QRHXD-H group exhibiting the most pronounced efficacy.

Moreover, to identify the unknown components of the herbal formula, we conducted LC-MS/MS analysis of QRHXD and found that it contained various bioactive compounds, including chlorogenic acid, sinomenine, paeoniflorin, calycosin-7-*O*-glucoside, astilbin, atractylodin, berberine hydrochloride, curcumin, and tanshinone IIA. Numerous studies have demonstrated that these components can alleviate disease activity and mitigate bone destruction in individuals with RA. For instance, chlorogenic acid has been shown to reduce B-cell activating factor (BAFF) and TNF-α levels, inhibit the apoptosis of MH7A cells, and slow the progression of arthritis in CIA mice (22). A review suggested that sinomenine and paeoniflorin can modulate endoplasmic reticulum stress to exert anti-inflammatory effects, making them potential therapeutic agents for RA (23). Further experiments demonstrated that sinomenine downregulates the Src/FAK/P130Cas signalling pathway to inhibit macrophage migration, thereby exerting anti-inflammatory effects (24). Paeoniflorin has been shown to regulate the balance of the inflammatory-immune system, contributing to the treatment of AIA in rats (25). Additionally, astilbin may demonstrate satisfactory efficacy against RA by activating the A2AAR/adenosine system and inhibiting ERK/nuclear factor-kappa B (NF-kB)/STAT signalling pathways (26). Atractylodin has been shown to significantly downregulate CD40, CD80, and CD86 in the spleen, inhibit dendritic cell activation and proinflammatory cytokine secretion, and reduce joint swelling in CIA mice (27). A meta-analysis conducted by Kou reported that curcumin supplementation improved inflammation levels and clinical symptoms in RA patients (28). Moreover, animal studies have demonstrated that curcumin can inhibit the activation of the PI3K/AKT signalling pathway, suppress TNF-α, IL-6, and IL-17 expression; and ameliorate joint inflammation in CIA rats (29). In our preliminary study on tanshinone IIA, we found that it could reduce the accumulation of reactive oxygen species (ROS), inhibit osteoclast (OC) differentiation, and mitigate bone loss in AIA rats (13).

As a key protein in the glycolysis/gluconeogenesis and AMPK signalling pathways, FBP1 influences OC differentiation through glucose metabolism (30) and plays a role in programmed cell death, which is closely linked to hypoxia, inflammation, immunity, and iron death (31, 32). FBP1 is a rate-limiting enzyme in the gluconeogenic pathway and is elevated during starvation. On one hand, it participates in gluconeogenesis to maintain blood glucose; on the other hand, it inhibits the insulin pathway, reducing lipid synthesis and sugar consumption. These dual functions are coordinated to integrate metabolic and signalling pathways at the molecular level (33). FBP1 regulates recombinant axis inhibition protein (AXIN) to influence AMPK signalling. Liver kinase B1 (LKB1), an initiator of AMPK, activates the downstream catabolic pathway by binding to vacuolar-type ATPase (v-ATPase) with AXINs on the lysosomal membrane. When extracellular glucose and intracellular FBP1 levels decrease, fructose-1,6-bisphosphatase (FBP) is activated, promoting the dynamic binding of v-ATPase and AXINs (34). Moreover, FBP can bind to transient receptor potential vanilloid (TRPV) channels, inhibiting calcium ion channel activity, which may ultimately activate the AMPK signalling pathway (35).

AMPK is a crucial pathway for glycolysis/gluconeogenesis, serving as a key regulator of metabolic homeostasis. When the body experiences hypoxia or energy deficiency, adenosine diphosphate (ADP) binds to the γ-subunit of AMPK, while LKB1 phosphorylates threonine 172 on the α-subunit, activating the AMPK pathway. This activation further phosphorylates downstream genes involved in glucose metabolism, lipid metabolism, transcription factor production, mitochondrial homeostasis, and cellular autophagy (36). Additionally, AMPK plays a role in the maturation and differentiation of OC and osteoblasts (OB), contributing to the regulation of RA-related bone destruction. AMPK is involved in osteoclast metabolism through immune signalling pathways such as NF-kB. Activated AMPK inhibits receptor activator of nuclear kappa-B ligand (RANKL)-induced OC differentiation by reducing the expression of the cellular oncogene Fos (c-Fos) and recombinant nuclear factor of activated T cells, cytoplasmic 1 (NFATc1) (37). Additionally, AMPK can silence Beclin1 (BCN1), induce cellular autophagy, and promote OB differentiation and angiogenesis (38).

QRHXD regulates the AMPK signalling pathway to treat RA by inhibiting FBP1. In this study, we found that FBP1 was significantly overexpressed in the serum of RA patients, accompanied by the downregulation of AMPK signalling, as revealed through a combined multiomics analysis of serum. This may be due to the increased energy consumption in RA patients, where glucose is insufficient to meet metabolic demands, leading to the active activation of FBP1 for glucose synthesis. In contrast, after treatment with QRHXD, the inflammatory state and immune disorders in RA patients were alleviated, reducing the body’s energy consumption and rendering FBP1 inactive.

## Conclusion

In conclusion, on the basis of proteomics, metabolomics, and animal experiments, this study demonstrated that QRHXD could reduce disease activity and the inflammatory response in RA patients while delaying bone destruction in CIA mice, by inhibiting FBP1 and activating the AMPK signalling pathway. This study provides new insight into the treatment of RA with QRHXD. However, it has several limitations. Although we identified the active ingredients of QRHXD, the mechanism underlying their effects on RA remains unclear. In the future, we will conduct drug target-molecule docking experiments with QRHXD to elucidate the “pharmacological component-disease molecule-signalling pathway” communication network of QRHXD against RA.

## Data Availability

The mass spectrometry proteomics data have been deposited to the ProteomeXchange Consortium (https://proteomecentral.proteomexchange.org) via the iProX partner repository with the dataset identifier PXD054129. The metabolomics data have been deposited to the OMIX database (https://www.cncb.ac.cn/) with dataset identifier OMIX006902.
